# The Vaginal Microbiome: Patient- versus Physician-Collected Microbial Swab: A Pilot Study

**DOI:** 10.3390/microorganisms12091859

**Published:** 2024-09-07

**Authors:** Xu Shan Gao, Thomas Groot, Sam Schoenmakers, Yvonne Louwers, Andries Budding, Joop Laven

**Affiliations:** 1 Division of Reproductive Endocrinology and Infertility, Department of Obstetrics and Gynecology, Erasmus University Medical Center, 3015 GD Rotterdam, The Netherlands; 2 InBiome B.V., 1098 XG Amsterdam, The Netherlands; 3 Division of Obstetrics and Fetal Medicine, Department of Obstetrics and Gynecology, Erasmus University Medical Center, 3015 GD Rotterdam, The Netherlands

**Keywords:** vaginal microbiota, reliability, patient-collected swabs, *Lactobacillus*, microbial analysis

## Abstract

The composition of the vaginal microbiota prior to an IVF/IVF-ICSI treatment can predict the chance of achieving a pregnancy. To improve clinical applicability and be more patient-friendly, the self-collection of vaginal samples would be preferable. However, the reliability of patient-collected samples compared to physician-collected samples remains unclear. This study compares microbiome outcomes from patient-collected versus physician-collected vaginal samples. This is a prospective pilot study consisting of two cohorts: Cohort I involved patient self-sampling of the vagina, followed by a physician-collected vaginal swab, while Cohort II involved the reversed order of collection. The interspace profiling (IS-Pro) technique was used to analyze the microbiota composition in all samples. From May 2021 to March 2022, a total of 444 samples were collected from *n* = 222 patients (aged 21–44 years), with Cohort I (*n* = 109) and Cohort II (*n* = 113). The vaginal microbiome composition of both cohorts was highly similar, regardless of the sampling order, with a mean cosine similarity of 0.93 (95% CI 0.91, 0.95) in Cohort I and 0.94 (95% CI 0.92, 0.96) in Cohort II. Furthermore, ANOVA analysis revealed no significant differences in bacterial species abundance between physician- and patient-collected samples, nor between first and second sample collections. The self-collection of vaginal samples can be considered comparable to physician-collected samples and indicates a more patient-friendly and convenient collection of the vaginal microbiome in an outpatient clinical setting.

## 1. Introduction

Over the last few decades, the role of the microbiome in human health and disease has been extensively investigated, and it has been shown to play an active and integrated role in human metabolism physiology and immune function. Focusing on the human vaginal microbiome, a healthy microbial composition consists of mainly *Lactobacillus* spp., with Lactobacilli exceeding over 70% abundance, whereas in other mammals, they rarely surpass 1%. Lactobacilli create an acidic vaginal environment by producing lactic acid, maintaining the pH balance of the vaginal ecosystem, and inhibiting the overgrowth of harmful microorganisms [1]. However, the total composition of the vaginal microbiota is highly dynamic and can be affected by the menstrual cycle, pregnancy, diet, lifestyle, ethnicity, contraceptive use, and age [2,3,4,5,6,7]. Lately, the role of the vaginal microbiota in reproductive health and outcome has gained attention [8]. Current research on reproductive outcomes indicates that the dominant presence of *Lactobacillus* (>90%) species within the female reproductive tract in general is linked to successful reproduction. Lower implantation rates and miscarriage appeared to be associated with a non-dominant *Lactobacillus* environment (<90%) with higher microbial diversity. Moreover, the latter was linked to adverse reproductive outcomes and various gynecological diseases, such as infertility, spontaneous abortion, pre-term birth, polycystic ovary syndrome, bacterial vaginosis, and sexually transmitted infections, including human papillomavirus (HPV) infection [9,10,11,12,13,14,15]. Studies have suggested that specific compositions of the vaginal microbiota may influence the local immune response and be involved in the occurrence of cervical cancer and the clearance of HPV infection [16]. The exact mechanisms by which microbiota influence the immune response still have to be elucidated but likely involve interactions between the microbiota, local immune responses, and viruses [16].

Understanding and maintaining a healthy vaginal microbiota, dominated by Lactobacillus species, is crucial for reproductive health and reducing the risk of gynecological diseases and cervical cancer linked to HPV infection.

Longitudinal studies are essential to understand the dynamics of the vaginal microbiota throughout physiological processes and in the development of diseases. However, these studies involve multiple clinical visits, presenting challenges for healthcare providers. To address this, our study aims to assess the reliability and accuracy of patient-collected vaginal swabs as a more patient-friendly and less time-consuming alternative to physician-collected samples. Although previous research has demonstrated the reliability of self-collected samples for specific bacteria causing reproductive tract infections and sexually transmitted infections, we aim to evaluate the feasibility of self-collected samples regarding the overall composition of the vaginal microbiome [17,18,19]. Current limitations in the literature arise from physician-collected samples being taken after patient-collected samples, which means any contamination caused by the patient would also be present in the physician-collected sample. To address these potential problems, we propose a method in which the patient-collected sample is first obtained in one cohort, whereas in another cohort, the patient-collected sample is obtained after the physician-collected sample. This approach ensures more reliable comparisons and helps to accurately assess the equivalence of microbiota compositions between patient-collected and physician-collected samples while also investigating the potential for contamination during self-collection.

## 2. Materials and Methods

### 2.1. Study Population

Patients at our fertility clinic were invited to participate either before the start of their IVF/IVF-ICSI treatment or on the day of the cryopreserved embryo transfer in a natural cycle. Patients were excluded if they had used antibiotics within 3 months prior to the start of their IVF or IVF-ICSI intake. Additionally, those undergoing IVF as an emergency treatment due to cancer were also excluded.

This study was conducted in accordance with the Declaration of Helsinki and approved by the Ethics Committee of Erasmus Medical Centre (The Netherlands) (protocol code MEC-2021-0223).

### 2.2. Study Design and Sample Collection

This prospective pilot study consisted of two cohorts. In Cohort I, the patient was asked to collect a vaginal swab after instructions on how to perform self-sampling. This was followed by a physician-collected swab without the use of a speculum, while the patient was seated in a gynecological examination chair. Cohort II involved the reversed order of collection: first, a physician-collected swab, followed by self-sampling. The patients collected the vaginal swabs without a physician present in the examination room. The second sample in both cohorts was collected immediately after the first, ensuring minimal time elapsed between the two samples. 

All swabs were collected using FLOQSwabs™ (Copan Italia SpA, Brescia, Italy), and all participants were instructed by the same researcher. Samples were collected on days when the patients already had an appointment at the clinic and were not menstruating. The following steps were explained verbally to the participants to ensure the instructions were clear: For the preparation, the participant was asked to open the cap of the tube and place this on a stable surface to avoid contamination during the procedure. This precaution was emphasized to prevent opening the cap after the sample had been collected, which could lead to contamination. For the positioning, the participants were advised to assume a comfortable position, similar to the position taken when inserting a tampon. Options included standing with one leg elevated on a stable surface (like a toilet seat or chair) or sitting with knees apart while standing or sitting. For the sampling technique, the patients were instructed to spread the labia with one hand, insert the swab 3–5 cm beyond the vaginal orifice with the other hand, and rotate the swab along the vaginal wall for 10–15 seconds. It is important to note that voiding and cleaning were not performed before swabbing. After collecting the sample, the swabs were immediately placed back into Eppendorf tubes filled with an eNAT^®^ medium, which was stored between 5 and 25 grades Celsius. The eNAT^®^ medium is a guanidine-thiocyanate-based medium specially designed to stabilize RNA and DNA of bacteria, which makes it possible to preserve for prolonged time periods at room temperature [20]. The samples were sent to the inBiome lab (Amsterdam, The Netherlands), arriving the next business day and being analyzed the day after. The instructions for use for patients with illustrations can be found in Supplementary S1.

### 2.3. DNA Extraction and Vaginal Microbiota Analysis

The amplification of the intergenic spaces (IS) regions was performed with the molecular culture assay, according to the protocol provided by the manufacturer (inBiome, Amsterdam, The Netherlands). Molecular culture was based on the IS-pro technique; a eubacterial assay was performed based on the detection of length polymorphisms of the 16S–23S rDNA gene IS region and sequence polymorphisms in the neighboring 16S rDNA.

Briefly, the procedure consists of two separate PCRs: The first PCR mixture contains two different fluorescently labeled forward primers targeting different bacterial groups and three reverse primers, thus providing universal coverage [21]. The first forward primer specifically targets the phyla Firmicutes, Actinobacteria, Fusobacteria, and Verrucomicrobia (FAFV), while the second labeled forward primer targets the phylum Bacteroidetes. A separate PCR mixture, specific for the phylum Proteobacteria, contains a labeled forward primer combined with seven reverse primers.

The amplifications were performed using a GeneAmp 9700 PCR system (Applied Biosystems, Foster City, CA, USA). After PCR, 5 μL of the PCR product was mixed with 20 μL of formamide and 0.2 μL of a custom size marker (inBiome, Amsterdam, The Netherlands). DNA fragment analysis was conducted on an ABI Prism 3500 genetic analyzer (Applied Biosystems, Foster City, CA, USA). Data were analyzed, and species were identified with the IS-pro proprietary software suite (inBiome, Amsterdam, The Netherlands, version 0.17.0). The results are presented as bacterial profiles and lists of the called species and their abundances. A brief glossary of the technical jargon related to DNA extraction and analysis is provided in Supplementary Table S1.

### 2.4. Cosine Distance and Comparison Analysis

To evaluate the similarity between vaginal microbiota profiles obtained from different sampling methods, comparisons were conducted at the species level using both SHAP (Shapley additive explanations) and raw IS profiles. The IS profiles, representing bacterial profiles, were structured as one-dimensional arrays where each position corresponds to the nucleotide length of the 16S–23S rDNA gene IS region of a bacterial species, and each value indicates the abundance of that species. 

#### 2.4.1. Cosine Similarity and Modification

To measure the similarity between these raw microbiota profiles, we employed a modified version of cosine similarity. This similarity measure was selected for its ability to capture both directional and magnitude differences in high-dimensional bacterial profiles, which is standard practice in vector mathematics.

In sequence length analysis with capillary electrophoresis (CE), slight deviations in measured nucleotide lengths can occur due to the noise inherent to the CE process. This noise can cause misalignment of peaks, resulting in artificially low similarity scores when using methods like cosine similarity. To address this issue, we developed a method to improve the alignment of nucleotide lengths by widening each peak with a sliding window of typically 3-nucleotide lengths, thereby enhancing the accuracy of similarity measurements.

Once the bacterial profiles were converted, cosine similarity between bacterial profiles was calculated using the SciPy library version 1.14.0 for Python version 3.10.12.

#### 2.4.2. Analysis of Bacterial Species Abundance Differences

To investigate the differences in bacterial species abundance between the two cohorts based on the sample collection method, we conducted a one-way ANOVA test. The null hypothesis assumed equal means between the cohorts. A *p*-value of less than 0.05 was considered significant.

To complement the ANOVA analysis and gain deeper insights into species-specific abundance patterns, we employed SHAP (Shapley additive explanations). A linear regression model was fitted to the data, and SHAP beeswarm plots visualized the impact of different bacterial species on the distinction between physician- and patient-collected samples. Additionally, the plots illustrated the influence of the sample collection method and order on species abundance. 

Similar to the cosine similarity method, bacterial profiles were presented using one-dimensional arrays, and a default linear regression model was fitted to predict the differences between sampling methods using the scikit-learn library version 1.5.1. Shapley values were estimated using the SHAP library version 0.45.1 for Python version 3.10.12, with the SHAP Explainer class automatically selecting the value estimation algorithm. 

### 2.5. Statistical Analysis

Statistical analyses of the data were performed by using SPSS statistics version 28 (IBM Corp, Armonk, NY, USA). To analyze the potential differences in bacterial species between physician- and patient-collected vaginal samples, SHAP plots were employed using Python version 3.10.12. The SHAP values were visualized using beeswarm plots, which were generated using the ‘shap’ library version 0.45.1. Univariate analyses were conducted to find the differences between the two cohorts. The chi-square test or Fisher’s exact test was used for the categorical data. The independent-sample *t*-test and Mann–Whitney U test were used for continuous data. Two-sided *p*-values less than 0.05 were considered statistically significant.

## 3. Results

**Scheme 1:**
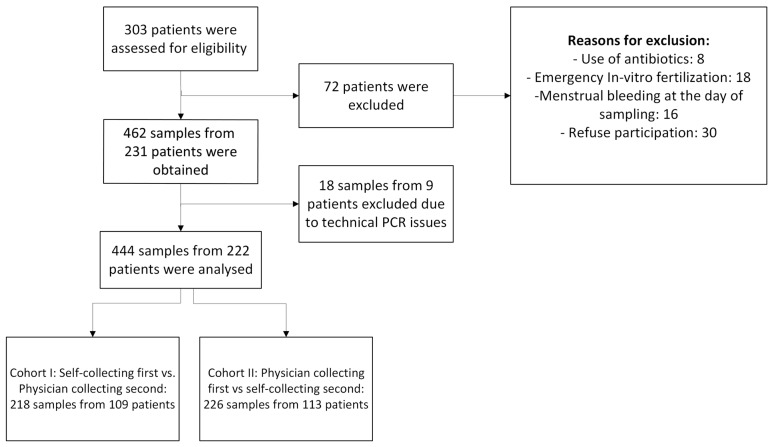
Flowchart of patient inclusion.

From May 2021 to March 2022, 303 patients were assessed for eligibility. However, 72 patients were excluded due to the use of antibiotics in the past three months (*n* = 8), emergency IVF treatment (*n* = 18), menstrual bleeding on the day of sampling (*n* = 16), or declining participation (*n* = 30). Limited time and perceived lack of personal benefit, given the concurrent IVF/ICSI treatment, were the primary reasons for patient decline. A total of 462 samples were collected from 231 patients (aged 21–44 years). Molecular culture failed for nine samples, due to variations in the PCR process, causing size markers to fall below the detection limit. Finally, 444 samples from 222 patients were available for the final analysis (*n* = 109 for Cohort I and *n* = 113 for Cohort II). See Scheme 1.

Table 1 shows the baseline characteristics for the two cohorts. The majority of included patients were of Western European descent (72%). None of the patients used hormones in the last month before sample collection. The median BMI in the two groups was similar (24.0 kg/m^2^). Univariate analyses showed no significant differences in BMI, age, or ethnicity between the two cohorts.

**Table 1:** Baseline characteristics of Cohort I (self-collection first) and Cohort II (physician collection first).

	Cohort I: Self-Collecting First vs. Physician Collecting Second (*n* = 109)	Cohort II: Physician Collecting First vs. Self-Collecting Second (*n* = 113)	*p*
BMI, kg/m^2^ (Median, range)	23.8 (18.4–33.9)	23.99 (17.7–35.6)	0.843
Age, years (SD)	34.9 (3.81)	34.7 (4.91)	0.956
IVF or IVF-ICSI population	68	74	0.696
Cryopreserved embryotransfer, natural cycle	41	39	
Previous attempts of IVF or IVF-ICSI			0.684
Yes	56	54	
No	52	56	
Indication for IVF of IVF-ICSI treatment			0.429
Male factor	64	53	
Combination	23	23	
Idiopathic	14	14	
Cycle disorder	3	12	
Uterine factor	1	4	
Tuba factor	1	6	
Other	3	1	
Ethnicity			0.06
Caucasian	80	80	
Mediterranean	12	4	
Hindu	2	5	
Asian	6	9	
African	7	13	
Latin American	2	2	

### 3.1. Comparison Analysis

#### 3.1.1. Similarity Score between Cohort I and II

The vaginal microbiome compositions of patient-collected and physician-collected vaginal samples were found to be highly similar, regardless of the order of sampling, with a mean cosine similarity of 0.93 (95% CI 0.91, 0.95) in Cohort I and 0.94 (95% CI 0.92, 0.96) in Cohort II, indicating that the samples were almost equivalent to each other. See Table 2.

High similarity in microbiome profile (cosine) between Cohort I (patient collecting first, physician after) and Cohort II (physician collecting first, patient after) with a 95% confidence interval. See the Methods section for the calculation of cosine similarity.

#### 3.1.2. SHAP Plots of Patient- and Physician-Collected Samples

**Figure 1:**
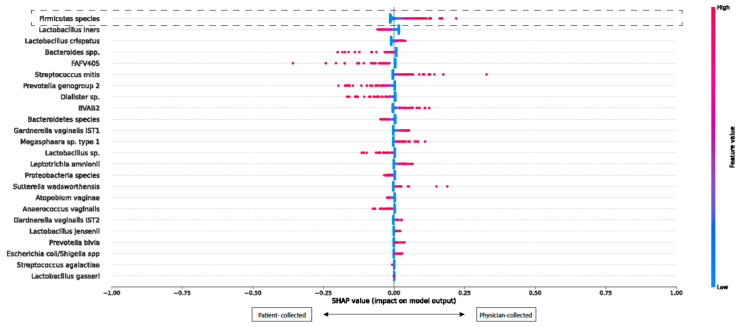
SHAP beeswarm plot showing the contributions of bacterial species in physician-collected and patient-collected vaginal samples.

The SHAP beeswarm plot visualizes the impact of different bacterial species, helping to interpret how each species influences the distinction between physician- and patient-collected samples. See Figure 1.

The mean SHAP values for most bacterial species cluster around zero, indicating no significant difference in their contribution regardless of whether the sample was collected by the physician or whether the patient themselves collected it. Furthermore, the contributions of various species were mixed, with no clear patterns emerging. Consequently, the differences in bacterial species are not attributed to self-collected or physician-collected samples but rather to a random distribution across samples. This conclusion was further supported by the one-way ANOVA test, which showed no significant differences in the bacterial species’ abundances between the physician- and patient-collected samples (see Supplementary Table S2). This supports the conclusion that contamination is unlikely, and the microbiota compositions are comparable.

#### 3.1.3. SHAP Plot of All First and All Second Collected Samples

**Figure 2:**
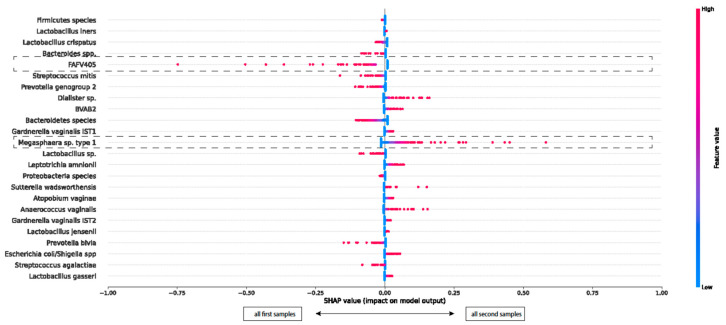
SHAP beeswarm plot: clustering of bacterial species for differentiation between first collected and second collected vaginal samples.

In addition, we investigated whether microbiome disturbance occurred after the first collected sample independently of who collected the first sample. *Megasphaera* sp. type 1 and *FAFV405* had higher SHAP values, indicating their association with the order of sampling. See Figure 2. Despite these findings, the overall pattern shows that the mean SHAP values for most species are distributed around zero. This SHAP plot indicates that the order in which samples were collected did not influence the microbiome composition. Again, the ANOVA test did not show any significant differences. See Supplementary Table S3.

#### 3.1.4. Summary

In summary, these results indicate that there is no concerted difference between patient-collected or physician-collected swabs. The analyses indicate the following:

- High cosine similarity: the microbiota compositions of patient-collected and physician-collected samples demonstrated a high degree of similarity;
- Uniform species distribution: no single bacterial species exhibited a preferential presence in either the patient-collected or physician-collected samples;
- Sampling sequence: the order in which samples were collected did not significantly influence the microbiome outcomes.

**Description of the SHAP beeswarm plot (Figure 1):** The SHAP beeswarm plot was used to illustrate the contribution of various bacterial species in distinguishing between physician-collected and patient-collected samples. 

X-axis: The SHAP values on the X-axis represent the contribution of each bacterial species to whether the sample was physician-collected or patient-collected. Positive SHAP values (toward the right) suggest that the bacterial species is more indicative of a physician-collected sample. Negative SHAP values (toward the left) suggest that the bacterial species is more indicative of a patient-collected sample.

Y-axis: The Y-axis lists the bacterial species included in the model. While SHAP values do not have an upper limit, a mean SHAP value of 1.00 indicates a relatively strong predictive association with physician-collected samples. The same interpretation applies to the negative SHAP values concerning patient-collected samples.

Color gradient: The color of each dot reflects the abundance of the bacterial species, ranging from blue (indicating low abundance) to red (indicating high abundance). Each colored dot corresponds to an individual sample. 

**Examples:**

In the case of the Firmicutes species, some samples exhibit positive mean SHAP values (toward the right), suggesting a higher abundance in physician-collected samples. 

Conversely, species like Lactobacillus crispatus and Bacteroidetes species show the mean SHAP values distributed around zero, indicating no significant association with either collection method. 

**Conclusion of the SHAP beeswarm plot (Figure 1):** the overall pattern shows that the mean SHAP values for most species are distributed around zero, with no species showing a strong and consistent pattern toward the physician-collected or patient-collected samples. This suggests that no single bacterial species significantly differentiates between physician- and patient-collected samples.

**Description of the SHAP beeswarm plot (Figure 2):** The SHAP beeswarm plot illustrates the influence of different bacterial species in distinguishing between the first and second collected samples.

X-axis: Positive SHAP values (toward the right) suggest that the bacterial species is more indicative of a second sample. Negative SHAP values (toward the left) suggest that the bacterial species is more indicative of a first collected sample. 

Y-axis: The Y-axis lists the bacterial species included in the model.

Color gradient: The color of each dot reflects the abundance of the bacterial species, ranging from blue (indicating low abundance) to red (indicating high abundance). Each colored dot corresponds to an individual sample. 

**Examples**:

Megasphaera sp. type 1 shows points with positive SHAP values up to 0.6 (toward the right), suggesting a stronger association with a second sample. 

Conversely, FAFV405 shows negative SHAP values up to 0.75 (to the left), indicating a stronger association with the first collected sample. 

**Conclusion of the SHAP beeswarm plot (Figure 2):** Although some samples show high SHAP values for certain species, the overall pattern shows that the mean SHAP values for most species remain close to zero. This SHAP plot indicates that the order in which samples were collected did not influence the microbiome composition.

## 4. Discussion

The present study demonstrated that the patient-collected vaginal swabs had a highly similar microbiota composition to physician-collected swabs. Furthermore, no single bacterial species significantly differentiated between physician- and patient-collected samples. Also, the order in which samples were collected did not significantly influence the vaginal microbiome composition. Therefore, the self-collection of vaginal samples is a reliable and patient-friendly alternative to physician collection at home or in outpatient clinics, as well as clinical settings.

### 4.1. Comparison of Previous Research 

Previous studies using the 16s rRNA next-generation sequencing techniques have also found that patient-collected vaginal swabs have the same microbial diversity as physician-collected swabs in non-pregnant women [22] as well as during pregnancy [23]. Previous studies that utilized a speculum during physician collection did not report any issues related to contamination [22,23]. Unlike previous studies, in this study, we chose to refrain from using a speculum during physician collection to create a more patient-friendly procedure. Moreover, despite the difference in positioning between the physician-collected method (with the patient in a gynecological examination chair) and patient collection (with the patient standing), no specific contaminants such as *Escherichia coli* that could potentially impact the results were observed in our study.

### 4.2. Impact of Sample Collection Techniques

The few variations in the composition of the vaginal microbiota between patient- and physician-collected samples can be attributed to the process of sample collection and laboratory techniques. Moreover, the issue of repeat sampling did not appear to compromise the integrity of the vaginal microbiome samples. Previous research has demonstrated that the vaginal microbiome remains consistent between patient-collected samples and multiple swabs taken by a physician from the lateral wall of the mid-vagina [22]. The current study findings further support that repeat sampling does not negatively affect the reliability of microbiome assessments.

### 4.3. Clinical Implications

These findings could have implications for clinical practice. Self-collected vaginal swabs can be integrated into routine care, offering significant benefits such as increased patient autonomy, privacy, and convenience [24]. This method is particularly advantageous for patients undergoing IVF/IVF-ICSI treatment, where reducing clinic visits can enhance comfort and reduce stress. Customizing clinical protocols to include self-collection options can optimize patient engagement, reduce the burden on healthcare providers, and potentially improve patient outcomes by ensuring more consistent and timely sample collection. 

### 4.4. Limitations and Potential Biases

Potential biases include selection bias, as this study was limited to patients undergoing IVF/IVF-ICSI treatment. However, previous studies have shown that samples collected by patients were also effective for the evaluation of clinical infections in asymptomatic pregnant women [23] and in third-trimester pregnant women [25]. We believe that with detailed instructions, these findings could be generalizable to broader populations.

Additionally, the involvement of a single researcher in sample collection did not allow for an inter-observer variability assessment among the physicians. Despite these potential variations, the high degree of similarity observed among samples from a diverse range of patients indicates robust reliability. 

### 4.5. Patient Perspectives and Practical Considerations

Despite familiarity with hospitals and medical procedures, there was initial hesitation and fear about self-sampling among the IVF population. This cautiousness stemmed from concerns about performing the procedure correctly, even though these patients regularly undergo medical procedures and transvaginal ultrasounds. However, despite initial caution, they demonstrated willingness when given clear instructions. This underscores the need for detailed guidance to empower patients in self-collection practices within healthcare settings. 

The patient collection of vaginal swabs has already been implemented in various gynecological areas, for example, in human papillomavirus (HPV) screening to increase screening coverage in the cervical screening program of the Netherlands. The value, applicability outside of a hospital setting, and increased health potential of patient self-collection became evident during the COVID-19 pandemic and may have contributed to compensating for the lower participation rates during this period [26]. Despite a significant decrease in participation rates in 2020 during COVID-19 (50%) compared to 2018/10 (57%), the utilization of self-collection doubled in 2020 (16%) compared to 2018/19 (8%, *p* < 0.001) [26]. Moreover, a randomized controlled trial showed that patient-collected vaginal swabs were not inferior to physician-collected samples in the clinical performance of human papillomavirus (HPV) testing in terms of the detection of cervical intraepithelial neoplasia of grade 2 or worse [27].

### 4.6. Future Research Directions

Future research should focus on investigating the impact of self-collection on patient outcomes, including detection rates of infections or microbial dysbiosis on clinical outcomes and overall patient satisfaction. Additionally, in longitudinal studies on vaginal microbiota dynamics, patient-collected vaginal swabs could increase patient participation and offer insights into microbiome fluctuations [6,28]. Collecting samples in various real-life situations and conditions allows for a more accurate representation of how physiological and lifestyle factors, such as the menstrual cycle, contraceptives, diet, and exercise, impact the vaginal microbiome. The ability to collect samples at home with clear instructions, coupled with the use of robust transport media like eNAT^®^ that preserve nucleic acids for up to four weeks at room temperature, could further extend the applicability of self-sampling in clinical practice [20]. 

## 5. Conclusions

In conclusion, patient-collected self-sampled vaginal swabs are reliable for the determination of the vaginal microbiome at home or in outpatient clinics, as well as clinical settings, particularly benefiting IVF/IVF-ICSI patients by enhancing convenience and privacy and reducing clinic visits. This method significantly enhances patient comfort and convenience, particularly for those who may find clinic visits challenging, time-consuming, or uncomfortable. Patient-collected samples are particularly useful in remote or rural areas where healthcare access is limited, providing a convenient alternative for timely diagnoses. They are also beneficial for patients requiring frequent monitoring, such as those suffering from recurrent genital tract infections, by enabling consistent data collection to track changes over time. By embracing and expanding the use of self-collection methods, healthcare providers can improve patient engagement, optimize resource use, and ensure that more individuals receive the care they need in a manner that respects their comfort and autonomy. 

## Figures and Tables

**Table 2:** Similarity between the patient- and physician-collected samples in both cohorts.

	Similarity (Cosine)	95% CI
Cohort I	0.93	0.91–0.95
Cohort II	0.94	0.92–0.96

## Data Availability

The raw data supporting the conclusions of this article will be made available by the authors upon request.

## Supplementary Materials

The following supporting information can be downloaded at: https://www.mdpi.com/article/10.3390/microorganisms12091859/s1. Supplementary S1: Instructions for use. Table S1: Brief glossary of terms from Section “2.3. DNA Extraction and Microbiota Analysis”. Table S2: *p*-values of one-way ANOVA test for bacterial abundance in patient-collected vs. physician-collected vaginal swabs. Table S3: *p*-values of one-way ANOVA test for bacterial abundance in first vs. second collected vaginal swabs..
