# Supplementary material for: The Vaginal Microbiome: Patient- versus Physician-Collected Microbial Swab: A Pilot Study
Source: Microorganisms. 2024 Sep 7;12(9):1859. doi: 10.3390/microorganisms12091859 (PMC11434400; doi:10.3390/microorganisms12091859)
Supplement: Supplementary file 1 [file microorganisms-12-01859-s001.zip › microorganisms-3161151-supplementary.pdf]

Supplementary S1. Instructions for use

EN: Instructions for use for patient

1. Place the tube filled with eNAT-buffer on a solid and stable surface
2. Open the cap of the tube, which is filled with eNAT-buffer
3. Open the peel and extract the swab, only hold the swab at the side without the tip
4. Spread the labia with one hand, so that the vagina is accessible for insertion of the swab
5. Insert the swab 3-5 cm into the vagina and rotate it 10-15 sec along the vaginal wall
6. Remove the swab from the vagina and break the swab at the breakpoint on the edge of the tube
7. Put the swab directly into the buffer; do not put the swab on a surface
8. Close the tube with the cap. Close the container firmly and do not under tighten
9. Give the kit to your doctor

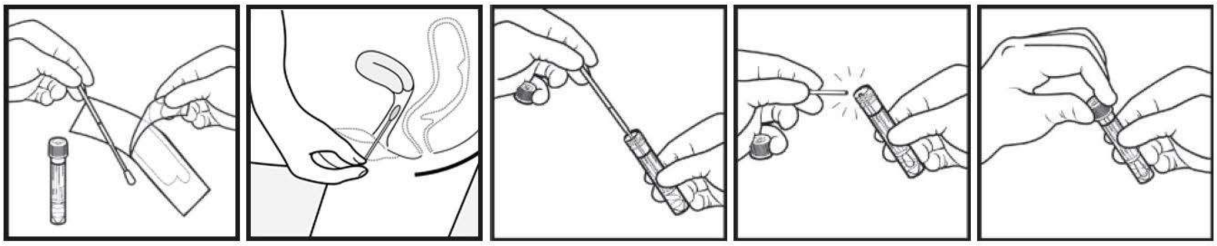

Supplementary Table S1: Brief Glossary of Terms from the paragraph "2.3. DNA extraction and microbiota analysis" (in alphabetical order):

**Term**	**Definition/explanation**
16S–23S rDNA Gene:	Regions of bacterial DNA commonly used in identifying and classifying bacteria.
Amplification	The process of creating multiple copies of a specific DNA segment, making it easier to study.
**DNA Extraction**	The process of isolating DNA from cells in a sample.
Eubacterial Assay:	A test designed to detect a broad range of bacteria by targeting universal bacterial DNA regions
Fluorescently Labeled Primers:	Primers that are tagged with a fluorescent dye, enabling the visualization and identification of amplified DNA fragments.
Formamide	A chemical used to prepare DNA samples for analysis by denaturing (unwinding) the DNA strands
Forward Primer	A short DNA sequence used to initiate DNA replication in PCR, specifically designed to target and bind to a certain DNA segment.
Fragment Analysis	A method to analyze DNA fragments based on their size, often used to identify different bacterial species.
Genetic Analyzer	A machine that sorts and measures DNA fragments, helping to identify different species based on the size and sequence of their DNA.
Intergenic Spaces (IS regions)	Sections of DNA located between genes, which can vary in length and sequence among different bacterial species.
IS-pro Technique	A method used to detect differences in DNA sequences and lengths between bacterial genes, specifically in the 16S–23S rDNA region.
Molecular Culture Assay	A method used to detect and quantify bacteria by amplifying specific DNA regions.
**PCR (Polymerase Chain Reaction)**	A technique used to make millions of copies of a specific DNA segment, making it easier to study.
Phyla	Broad classifications or groups of related bacteria.
Polymorphisms	Variations in DNA sequences or lengths among different individuals or species.
Reverse Primer	Another short DNA sequence used in PCR to complement the forward primer, allowing for complete DNA amplification.

Supplementary Table S2 *P*-values of one-way ANOVA test for bacterial abundance in patient-collected vs. physician-collected vaginal swabs

**Bacteria**	***p*- value**
Anaerococcus vaginalis	0.682993
Atopobium vaginae	0.154090
BVAB2	0.306357
Bacteroides spp.	0.592156
Bacteroidetes species	0.830372
Dialister sp.	0.790864
Escherichia coli/Shigella spp	0.870307
FAFV405	0.403861
Firmicutes species	0.576592
Gardnerella vaginalis IST1	0.200909
Gardnerella vaginalis IST2	0.370267
Lactobacillus crispatus	0.711948
Lactobacillus gasseri	0.999980
Lactobacillus iners	0.261802
Lactobacillus jensenii	0.241142
Lactobacillus sp.	0.582078
Leptotrichia amnionii	0.276940
Megasphaera sp. type 1	0.526664
Prevotella bivia	0.267190
Prevotella genogroup 2	0.368776
Proteobacteria species	0.283655
Streptococcus agalactiae	0.338432
Streptococcus mitis	0.163587
Sutterella wadsworthensis	0.813982

Supplementary Table S3 *P*-values of one-way ANOVA test for bacterial abundance in first vs. second collected vaginal swabs

**Bacteria**	***p*-value**
Anaerococcus vaginalis	0.528843
Atopobium vaginae	0.736777
BVAB2	0.797912
Bacteroides spp.	0.563007
Bacteroidetes species	0.932378
Dialister sp.	0.536629
Escherichia coli/Shigella spp.	0.785601
FAFV405	0.878863
Firmicutes species	0.789046
Gardnerella vaginalis IST1	0.645631
Gardnerella vaginalis IST2	0.976199
Lactobacillus crispatus	0.836166
Lactobacillus gasseri	0.974008
Lactobacillus iners	0.948476
Lactobacillus jensenii	0.967929
Lactobacillus sp.	0.733197
Leptotrichia amnionii	0.905826
Megasphaera sp. type 1	0.786287
Prevotella bivia	0.793408
Prevotella genogroup 2	0.706262
Proteobacteria species	0.971129
Streptococcus agalactiae	0.679399
Streptococcus mitis	0.836109
Sutterella wadsworthensis	0.766138
